# Cannabinoid Hyperemesis Syndrome, 2016 to 2022

**DOI:** 10.1001/jamanetworkopen.2025.45310

**Published:** 2025-11-24

**Authors:** James A. Swartz, Dana Franceschini

**Affiliations:** 1 Jane Addams College of Social Work, University of Illinois Chicago, Chicago

## Abstract

**Importance:**

Cannabinoid hyperemesis syndrome (CHS), ie, recurrent emesis from chronic cannabis use, is increasingly observed in emergency departments (EDs), yet population-level estimates and sociodemographic correlates remain underexplored.

**Objective:**

To estimate CHS prevalence in US EDs, assess trends from 2016 to 2022, and examine sociodemographic associations.

**Design, Setting, and Participants:**

This cross-sectional study, using data from the Nationwide Emergency Department Sample (approximately 85% of annual US ED visits) examined patients aged 12 years and older. Patients with ED visits related to pregnancy, gastroparesis, or chemotherapy were excluded.

**Main Outcomes and Measures:**

ED visits were grouped into 4 categories. Primary outcome was comparative temporal trends between each of the 4 categories: (1) no cannabis use or cyclic vomiting syndrome (CVS); (2) cannabis-related condition only; (3) CVS only; and (4) both cannabis-related condition and CVS (CHS proxy). Restricted cubic splines modeled temporal trends; multinomial logistic regression identified sociodemographic factors associated with CHS.

**Results:**

The analytic sample included 188 610 906 unweighted visits (806 million weighted); 433 251 323 (54.4%) were female; mean (SD) age, 47.9 years (95% CI, 47.8-48.1 years). CHS increased from 4.4 per 100 000 visits, peaked at 33.1 in quarter 2 2020, and remained elevated at 22.3 in 2022; concurrently, CVS-only declined from 300 to 186 per 100 000 visits. Females had lower CHS risk (RRR, 0.92; 95% CI, 0.88-0.95) as did patients in the South vs Northeast (RRR, 0.46; 95% CI, 0.40-0.53); risk was highest at ages 18 to 25 years (RRR, 3.59; 95% CI, 3.38-3.81) and 26 to 35 years (RRR, 2.26; 95% CI, 2.14-2.40).

**Conclusions And Relevance:**

In this cross-sectional study of US ED visits, CHS prevalence increased sharply during the COVID-19 pandemic and remained elevated. The findings highlight the need for continued vigilance and refinements to the clinical recognition of CHS.

## Key Points

**Question:**

How did cannabinoid hyperemesis syndrome (CHS) present and change in US emergency departments (EDs) from 2016 to 2022?

**Findings:**

In this cross-sectional study of the Nationwide Emergency Department Sample, with more than 188 million ED visits, CHS visits clustered among adults aged 18 to 35 years, rose during the COVID-19 pandemic from 2020 to 2021, then plateaued. Among ED visits with primary cyclic vomiting syndrome, the conditional probability of CHS remained elevated in 2022.

**Meaning:**

These findings suggest that CHS represents a growing share of emesis-related ED visits, especially among younger adults; greater awareness, standardized diagnostic practices, and use of the recently added *International Statistical Classification of Diseases and Related Health Problems, Tenth Revision (ICD-10) *codes (F12.188) may improve recognition and management as cannabis use expands postlegalization.

## Teaser

This cross-sectional analysis examines US emergency department data from 2016 to 2022 to investigate sociodemographic associations in cannabinoid hyperemesis syndrome.

## Introduction

As of June 2025, nearly half of all US residents lived in states with legalized recreational cannabis.^1^ The rapid and broad-scale changes in cannabis legality have been closely monitored by public health researchers, epidemiologists, and addiction researchers to evaluate whether legalization has produced adverse health outcomes.^2,3,4,5,6,7,8,9,10,11,12^ Evidence to date remains modest and mixed.^2,13^

Among cannabis-related harms, cannabinoid hyperemesis syndrome (CHS) has emerged as a growing public health concern.^14,15,16,17^ First identified in 2004 in Australia,^18,19^ the syndrome has uncertain etiology but several neurophysiological mechanisms have been proposed, including downregulation of high cannabinoid 1 receptors, altered transient receptor potential vanilloid 1 signaling, and disrupted hypothalamic thermoregulation.^20,21^ Genetic susceptibility has also been suggested, but evidence remains limited.^22^ In contrast, explanations attributing CHS to nonspecific gastrointestinal syndromes or environmental contaminants are not supported by scientific evidence.^23^

CHS presents with recurrent severe nausea and emesis, often with abdominal pain and compulsive hot bathing.^24,25^ Risk increases with prolonged use (>6 months), use of high potency products (eg, vapes), and daily or near-daily use.^26,27^ Patients with CHS often present repeatedly to emergency departments (EDs) but are frequently misdiagnosed with cyclic vomiting syndrome (CVS) or other nonspecific gastrointestinal conditions; some clinicians believe that these patients are exaggerating their symptoms for another purpose.^28,57^ When diagnosed, CHS has typically been inferred from a CVS diagnosis (*International Statistical Classification of Diseases and Related Health Problems, Tenth Revision *[*ICD-10*] code R11.15) or nonspecific emesis (*ICD-10* code R11) accompanied by a cannabis-related diagnosis such as cannabis abuse (*ICD-10* code F12.1) or poisoning (*ICD-10* code T40.711). A dedicated CHS code (F12.188) only became available with the 2025 *ICD-10* update.^29^ Cannabis use is frequently underreported in survey contexts and likely in clinical settings such as the ED.^30^ Limited studies comparing verified substance use with ED diagnoses suggest underreporting, leading to misdiagnosis and inadequate treatment.^31,32,33^

Despite diagnostic challenges, several studies have assessed CHS trends, though most used single-state data or restricted the sample to youths and young adults.^14,17,34^ To the best of our knowledge, only 2 national studies of CHS trends in ED visits exist. One study, using inpatient data (2005-2014), found the share of CVS patients with cannabis use rose from 2.2% to 21.2%.^35^ Another, using ED data (2006-2013), reported an increase in visits with co-occurring emesis and cannabis use from 2.3 to 13.3 per 100 000.^36^ Overall, research shows that since the early 2000s, CHS cases have been increasing.^14,37,38,39^

Both national studies relied on pre-2015 data, preceding widespread legalization, medical expansion, and decriminalization of cannabis in many states.^1^ Adult-use cannabis legalization expanded markedly during the 2016 to 2022 period. After the 2016 elections, roughly one-fifth of the US population lived in states where recreational use was legal; by November 2022, following Maryland and Missouri referenda (21 states and the District of Columbia), more than 155 million people, nearly half of Americans, lived in adult-use states.^1^ Given these policy shifts, changes in CHS prevalence since 2015 are plausible and merit updated analysis. This study updates national CHS prevalence estimates from 2016 to 2022 and examines whether CHS increased in tandem with cannabis-related diagnoses.

## Methods

### Overview

This cross-sectional study used data from the Nationwide Emergency Department Sample (NEDS), part of the Healthcare Cost and Utilization Project (HCUP), sponsored by the Agency for Healthcare Research and Quality.^40^ NEDS comprises a stratified 20% sample of all visits to hospital-owned EDs in participating hospitals across the United States. It includes deidentified information on patient demographics, treating hospital region, and up to 40 *ICD-10* diagnosis and procedure codes per visit.^41^ NEDS also provides design variables to account for stratification and sampling probabilities. This report follows Strengthening the Reporting of Observational Studies in Epidemiology (STROBE) reporting guidelines for cross-sectional studies. (eMethods in Supplement 1).

We analyzed NEDS data for 2016 through 2022. During this period, unweighted ED visit counts ranged from 28.3 million (2020) to 35.8 million (2018), with weighted national estimates ranging from 123.3 million (2020) to 144.8 million (2016 and 2017). Each year, data were contributed by 37 to 41 states. In 2022, for example, 993 hospitals across 41 states and the District of Columbia contributed data, representing approximately 84.7% of the US population and 83.9% of all ED visits.^40^ Prior years were similarly representative. Although not all states contribute data to NEDS in every year, the use of HCUP-provided survey weights allows for nationally representative estimates of ED visits.^42^

This study used deidentified data from the HCUP-NEDS database and was not considered human subjects research by the University of Illinois Chicago institutional review board. Therefore, informed consent was not required.

### Participants

The 2016 to 2022 NEDS dataset contained an unweighted total of 225.6 million ED visits. Because NEDS data captures visits rather than individuals, patients with multiple ED visits can appear multiple times. To construct the analytic sample, we excluded all visits involving children 0 to 11 years (n = 28.9 million) and visits with missing age (n = 9225). Although CVS can occur in chidren,^43^ CHS is rare in this age group, and most cannabis-related pediatric ED visits involve accidental ingestion or poisoning.^44,45,46^

To reduce diagnostic ambiguity, we excluded visits where CVS was the primary diagnosis but another *ICD-10 *code in the record suggested an alternative cause for emesis. These included gastroparesis (*ICD-10 *code K31.84; n = 551 407); migraine-related cyclical vomiting (G43.A1, n = 27 218); pregnancy-related nausea and emesis (*ICD-10 *code O21.X; n = 7 094 287); chemotherapy-related nausea (*ICD-10 *code T451X5A; n = 343 209); and bulimia nervosa (*ICD-10 *code F50.2; n = 14 946). We also excluded visits that involved any combination of these diagnoses (n = 10 467). After applying both age and diagnostic exclusions, the unweighted analytic sample included 188 610 906 ED visits.

### Measures

#### Cannabinoid Hyperemesis Syndrome

Because no specific *ICD-10* code for CHS existed during the study period, we applied diagnostic criteria to estimate the presence of cannabis use disorder (CUD), cyclic vomiting syndrome (CVS), and CHS for each ED visit. As used here, CUD refers to cannabis-related diagnoses—including use, abuse, dependence, and poisoning—and does not align precisely with *Diagnostic and Statistical Manual of Mental Disorders* (Fifth Edition)–defined CUD.

CUD was identified using the Clinical Classifications Software Refined (CCSR), which aggregates over 70 000 *ICD-10* codes into 530 clinically meaningful categories.^47^ We classified CUD as present if any diagnosis in the record fell under CCSR category MBD019 (cannabis-related disorders) or MBD30 (subsequent encounters for cannabis-related disorders). Because cannabis use is often underreported in clinical settings,^48,49^ this broader definition was used to optimize detection.

CVS was classified as present when the primary diagnosis (ie, the main reason for the ED visit) was either *ICD-10* code R11.15 (cyclical vomiting, not associated with migraine) or R11.10 (vomiting, unspecified). On the basis of these criteria, visits were assigned to 1 of 4 mutually exclusive groups: (1) no CVS, CUD, or CHS; (2) CUD only; (3) CVS only; and (4) CHS, defined as a primary CVS diagnosis with a cannabis-specific secondary diagnosis (F12x or T40.7Xx).

##### Covariates

Time trends were captured using calendar quarters from quarter 1 (Q1) 2016 through Q4 2022. These were modeled both categorically (28 binary indicators) and continuously (coded 1 to 28) depending on the analytic approach. Additional covariates included US census region (Northeast, Midwest, South, and West),^50^ biological sex (male or female), and age at admission, modeled as continuous and as categorial. Race and ethnicity was from HCUP’s uniform variable (patient-reported at registration when available or abstracted from the medical record). In NEDS, ethnicity supersedes race when both are provided. Categories were: American Indian or Alaska Native, Asian or Pacific Islander, Black, Hispanic, White, and other. HCUP uses other to include additional or multiple races and includes no further information. Race and ethnicity were added to the NEDS data beginning in 2019 and were available only for the 2019 to 2022 period. Disposition following the ED visit was included as a 5-category outcome: (1) treated and released; (2) admitted to the same hospital; (3) transferred to a short-term hospital; (4) died in the ED; or (5) not admitted, with an unknown postrelease destination.

### Statistical Analysis

All analyses were conducted using Stata version 19.5 (StataCorp LLC), employing the survey command set to account for NEDS sampling design, including clustering by facility and stratification and using Taylor series linearization.^51,52^ Analyses used 2-sided tests (α = .05). Descriptive statistics for covariates were calculated using survey-weighted data, disaggregated by CHS grouping. Bivariable comparisons across CHS groups were conducted using design-adjusted *F* statistics (Rao-Scott correction) for categorical variables and ordinary least squares regression for age, analyzed as a continuous variable. In multivariable models, age was included categorically in 5 groupings: ages 12 to 17, 18 to 25, 26 to 35, 36 to 50, 51 to 64, and 65 years or older.

We modeled temporal trends with logistic regressions predicting CHS, CVS, or CUD diagnoses; subgroup models stratified by sex, age, race and ethnicity, or region included quarter-by-demographic interactions, and marginal probabilities were plotted. We modeled calendar time with a restricted cubic spline (knots at Q4 2017, Q3 2019, Q2 2020 [COVID-19 onset], and Q1 2022). Sensitivity analyses with alternative knot placements and fixed-effects models yielded similar trajectories (eTable and eFigure in Supplement 1).

Finally, we used multinomial logistic regression (2019-2022) to identify demographic characteristic and clinical factors associated with CHS, CVS, and CUD, with visits lacking these conditions as the reference. Factors were entered simultaneously. The multinomial models included terms to assess both linear (quarter) as well as quadratic (quarter squared) trends.

## Results

The analytic sample included 188 610 906 unweighted ED visits, representing an estimated 806 million visits nationwide; 433 251 323 (54.4%) involved female patients, 85 515 094 (19.9%) involved patients who idetified as Black, 65 117 040 (15.1%) involved patients who identified as Hispanic, 252 810 185 (58.7%) involved patients who identified as White, and the median age was 48.0 years (95% CI, 47.8-48.1 years). Descriptive analyses revealed significant demographic and visit outcome differences across diagnostic groups (Table 1). Patients with CHS had a significantly younger mean (SD) age (30.6 [11.6] years) than those with CUD only (36.7 [15.0] years), CVS only (37.6 [19.7] years), or neither diagnosis (48.1 [21.2] years) (*P* < .001). Age categorization further confirmed that CHS patients were disproportionately represented in the 18 to 25 years (35.7%) and 26 to 35 years (31.5%) age groups compared with additional diagnostic groups (design-adjusted Wald-test, *P* < .001).

**Table 1.** Descriptive Statistics for Emergency Department Visits by Diagnostic Group (2016-2022)

Characteristics^a^	Without CUD, CVS, or CHS, No. (%)	CUD only, No. (%)	CVS only, No. (%)	CHS, No. (%)	*P* value
Region					
Northeast	145 219 530 (18.3)	2 255 743 (20.1)	317 862 (19.2)	26 757 (23.5)	<.001
Midwest	178 912 677 (22.6)	2 578 036 (23.0)	341 800 (20.6)	25 797 (22.7)	
South	316 547 981 (39.9)	4 031 655 (35.9)	646 439 (39.0)	30 755 (27.0)	
West	152 124 935 (19.2)	2 361 656 (21.0)	350 531 (21.2)	30 436 (26.8)	
Sex					
Male	359 473 140 (45.3)	7 084 884 (63.1)	649 025 (39.2)	55 950 (49.2)	<.001
Female	433 251 323 (54.7)	4 140 641 (36.9)	1 007 522 (60.8)	57 789 (50.8)	
Race and ethnicity					
American Indian or Alaska Native	2 788 166 (0.6)	58 019 (0.9)	6302 (0.8)	764 (0.8)	
Asian and Pacific Islander	9 899 037 (2.3)	73 212 (1.1)	17 191 (2.1)	1032 (1.1)	<.001
Black	85 515 094 (19.9)	2 030 278 (30.5)	176 955 (21.2)	22 700 (24.7)	
Hispanic	65 117 040 (15.1)	872 007 (13.1)	137 862 (16.5)	13 894 (15.1)	
Other^b^	14 421 862 (3.3)	229 638 (3.4)	29 086 (3.5)	3140 (3.4)	
White	252 810 185 (58.7)	3 400 427 (51.0)	466 026 (55.9)	50 366 (54.8)	
Age category, y					
12-17	48 453 794 (6.1)	601 582 (5.4)	203 772 (12.3)	6619 (5.8)	<.001
18-25	91 444 247 (11.5)	2 530 851 (22.5)	375 836 (22.7)	40 571 (35.7)	
26-35	124 325 137 (15.7)	2 927 046 (26.1)	354 057 (21.4)	35 867 (31.5)	
36-50	167 037 243 (21.1)	2 810 620 (25.0)	307 240 (18.5)	21 875 (19.2)	
51-64	160 669 980 (20.3)	1 842 705 (16.4)	210 679 (12.7)	7207 (6.3)	
≥65	200 836 416 (25.3)	513 834 (4.6)	204 869 (12.4)	1606 (1.4)	
Age, mean (SD), y	48.1 (21.2)	36.7 (15.0)	37.6 (19.7)	30.6 (11.6)	<.001
ED outcome					
Treat and release	652 524 469 (82.3)	6 877 349 (61.3)	1 623 178 (98.0)	101 210 (89.0)	<.001
Admitted to same hospital	123 564 971 (15.6)	4 208 540 (37.5)	14 298 (0.9)	12 003 (10.6)	
Transfer to short-term hospital	14 226 021 (1.8)	128 732 (1.1)	13 370 (0.8)	434 (0.4)	
Died in ED	1 407 871 (0.2)	3007 (0.0)	191 (0.0)	5 (0.0)	
Not admitted, unknown destination	1 081 791 (0.1)	9461 (0.1)	5596 (0.3)	93 (0.1)	

Abbreviations: CHS, cannabinoid hyperemesis syndrome; CUD, cannabis use disorder; CVS, cyclic vomiting syndrome; ED, emergency department.

^a^ Demographic characteristics (sex, race and ethnicity, age group, insurance status, and region) and ED outcomes are shown for patients with CHS and comparison groups. The analytic sample is restricted to ages 12 years and older (see Methods). Statistical comparisons use design-adjusted *F* tests accounting for survey stratification and clustering at the facility level.

^b^ The Healthcare Cost and Utilization Project breaks race and ethnicity into American Indian or Alaska Native, Asian or Pacific Islander, Black, Hispanic, Other, and White. Other includes additional or multiple races and includes no further information.

Among CHS visits (age ≥12 years), cannabis-specific diagnoses (F12.x or T40.7Xx) appeared high in the diagnosis array: the first cannabis code occurred as the second diagnosis in 52 534 (46.2%) of visits, the third in 23 744 (20.9%), and fourth in 14 261 (12.5%) of visits. Overall, CUD appeared in the second through fourth position in the diagnostic array in the 90 538 visits (79.7%) and in the second through sixth position in 103 635 (91.1%). Co-occurring noncannabis substance codes were uncommon: alcohol, 4094 (3.57%); opioids, 1879 (1.58%); cocaine, 2017 (1.77%); stimulants, 1254 (1.10%) (any of these, 7.21%), supporting the specificity of the CHS classification.

Sex differences were also observed. A slight majority of CHS patients were female 57 789 (50.8%). CVS-only and neither-condition patients were more likely to be female (1 007 522 [60.8%] and 433 251 323 [54.7%], respectively). The near-even sex distribution in CHS cases was distinct from the male predominance 7 084 884 (63.1%) observed for CUD and the female predominance observed in CVS (global design-adjusted Wald test *F*_1.49,5914.83_; *P* < .001).

Regional distributions differed significantly across diagnostic groups . CHS cases had higher relative shares in the West (30 436 [26.8%]) and Northeast (26 757 [23.5%]) compared with the South, which accounted for the largest overall share of ED visits (316 547 981 [39.9%]) (global design-adjusted Wald test, *P* < .001). These distributions differed from those observed in the other diagnostic groups, particularly the reference group without cannabis-related or CVS diagnoses.

Visit outcomes also varied significantly across diagnostic groups. Patients with CUD-only had the highest proportion of admissions to the same hospital (4 208 540 [37.5%]), whereas nearly all patients with CVS-only (1 623 178 [98.0%]) and the majority in the other groups were treated and released (global design-adjusted Wald test, *P* < .001).

Race and ethnicity distributions differed significantly across diagnostic groups. Black patients accounted for a larger share of CUD-only visits (2 030 278 [30.5%]) compared with CHS visits (22 700 [24.7%]) (global design-adjusted Wald test, *P* < .001), while distributions across additional racial and ethnic groups were relatively similar across diagnostic categories.

### Temporal Trends in Cannabinoid-Related and Vomiting Syndromes

**Figure 1.**
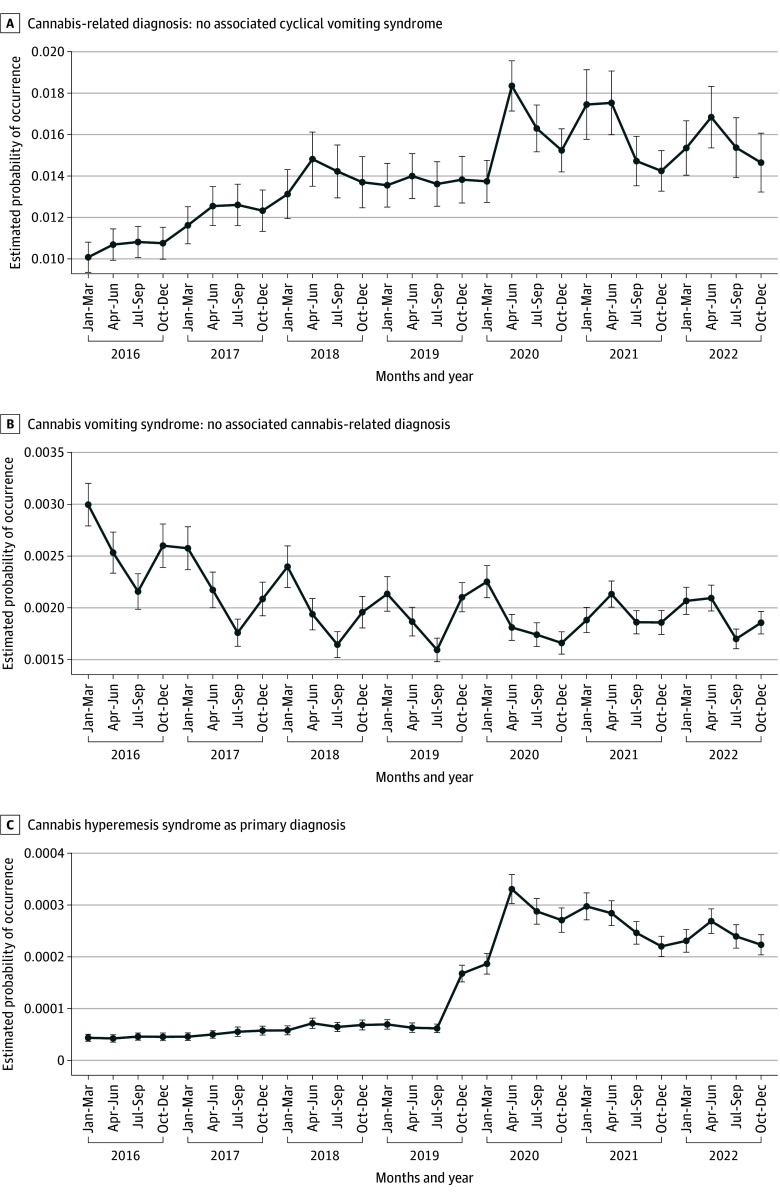
Emergency Department Visits With Cannabis-Related Diagnoses, Cyclic Vomiting Syndrome, and Cannabinoid Hyperemesis Syndrome, 2016 to 2022 Data source: Nationwide Emergency Department Sample, 2016 to 2022. Sample restricted to age 12 years or older; visits with pregnancy-related emesis, migraine, gastroparesis, gastrointestinal surgery, or bulimia excluded. Cannabinoid hyperemesis syndrome defined as a primary vomiting diagnosis (*International Statistical Classification of Diseases and Related Health Problems, Tenth Revision *[*ICD-10*] codes R11.10 or R11.15) with a cannabis-specific secondary diagnosis (*ICD-10 *codes F12x or T40.7Xx). Cyclic vomiting syndrome shows modest seasonality with lower rates in quarter 3 (July to September). Survey weights applied.

Temporal trends in ED visit diagnoses revealed distinct patterns across the three conditions (Figure 1). Between 2016 and 2022, CUD-only increased from 1008 to 1465 per 100 000 ED visits; CHS rose from 4.36 to 22.33 per 100 000 (peaking at 33.06 in Q2 2020); whereas CVS-only declined (300 to 186 per 100 000). This divergence suggests that the observed increase in CHS may be attributable to a growing subset of patients with CUD presenting with vomiting symptoms, rather than an overall rise in CVS diagnoses unrelated to exposure.

**Figure 2.**
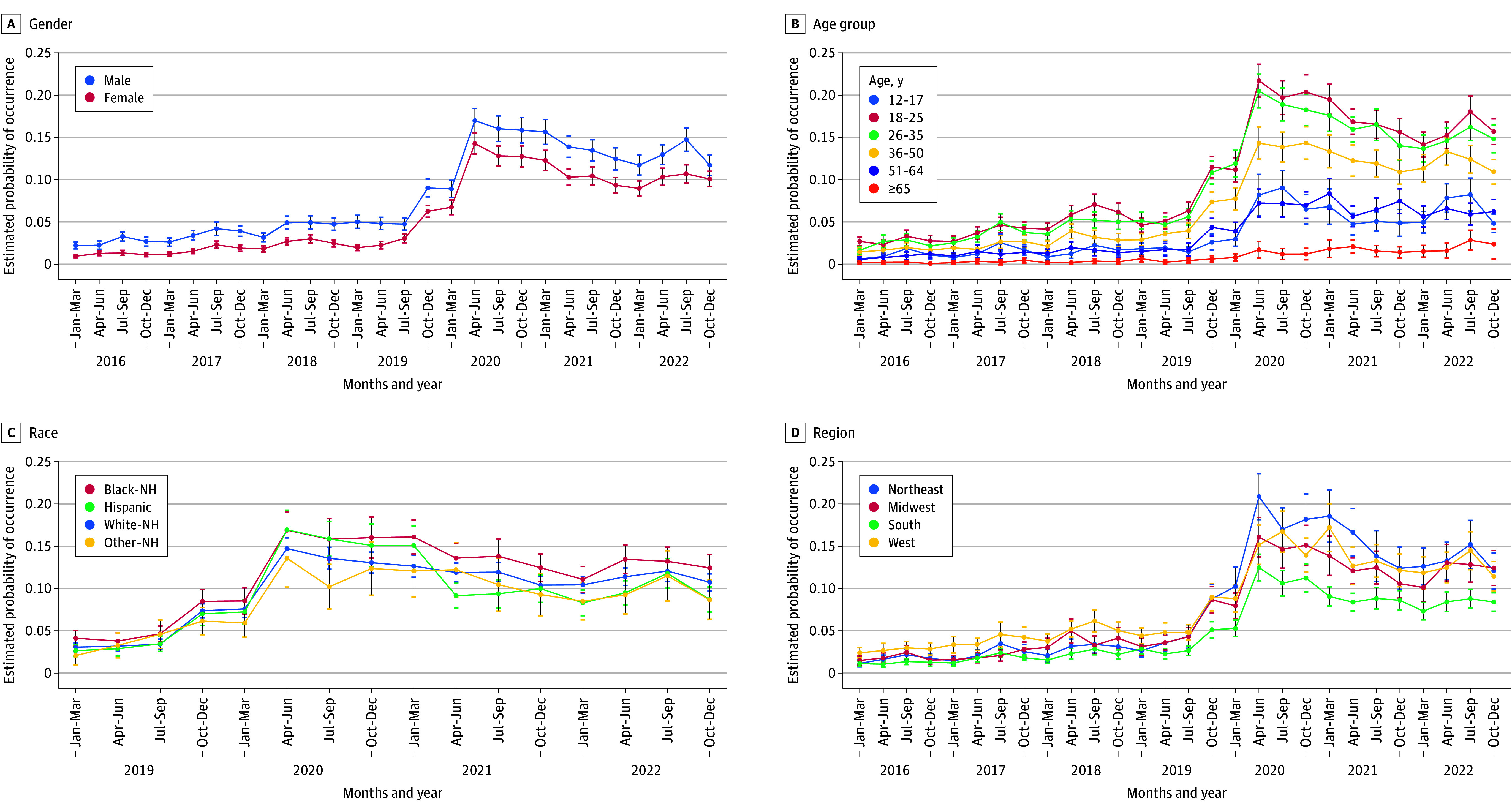
Cannabinoid Hyperemesis Syndrome by Demographic Group, 2016 to 2022 Data source: Nationwide Emergency Department Sample, 2016 to 2022. Analyses restricted to emergency department visits with a primary cyclic vomiting syndrome diagnosis; panels display survey-weighted quarterly estimated probability of cannabinoid hyperemesis syndrome by sex, age group, race and ethnicity, and region (race and ethnicity available 2019 to 2022). Sample restricted to age 12 years or older; visits with pregnancy-related emesis, migraine, gastroparesis, gastrointestinal surgery, or bulimia excluded. Cannabinoid hyperemesis syndrome defined as a primary vomiting diagnosis (*International Statistical Classification of Diseases and Related Health Problems, Tenth Revision *[*ICD-10*] codes R11.10 or R11.15) with a cannabis-specific secondary diagnosis (F12x or T40.7Xx). Survey weights applied. NH indicates non-Hispanic.

To further explore this shift, we examined changes over time in the probability of receiving a CHS diagnosis among ED visits coded with a primary diagnosis of CVS, stratified by demographic characteristics (Figure 2). These models were restricted to visits with a CVS code and estimated the conditional probability of CHS given a CVS diagnosis. Across all demographic groups, the probability of CHS increased over time, with sharp rises among those aged 18 to 25 and 26 to 35 years; estimates for ages 12 to 17 years were low and relatively stable. Male patients showed slightly higher CHS probabilities than female patients, while differences by race and ethnicity and region were more modest. These findings suggest evolving clinical practices or heightened awareness of CHS over time, particularly among specific demographic subgroups.

**Figure 3.**
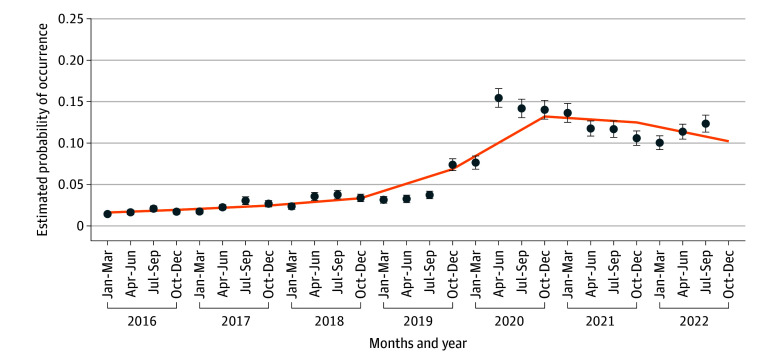
Conditional Probability of Cannabinoid Hyperemesis Syndrome Among Visits With Cannabis-Related Diagnoses, 2016 to 2022 Data source: Nationwide Emergency Department Sample, 2016 to 2022. Sample restricted to age 12 years and older; visits with pregnancy-related emesis, migraine, gastroparesis, gastrointestinal surgery, or bulimia excluded. Cannabinoid hyperemesis syndrome (CHS) defined as a primary vomiting diagnosis (*International Statistical Classification of Diseases and Related Health Problems, Tenth Revision *[*ICD-10*] codes R11.10 or R11.15) with a cannabis-specific secondary diagnosis (*ICD-10 *codes F12.x or T40.7Xx). Conditional probabilities *P*(CHS given primary cyclic vomiting syndrome emergency department visit) were estimated using survey-weighted logistic regression with a restricted cubic spline for calendar quarter (prespecified knots); points show quarterly survey-weighted estimates with 95% CIs and the line shows the spline fit.

Smoothed estimates of the quarterly probability of CHS among ED visits with primary CVS rose after Q2 2020, peaked in 2021, and tapered through 2023 (Figure 3); the empirical quarterly estimates with 95% CIs track the spline fit. The spline model demonstrated a statistically significant nonlinear increase in CHS estimated probability over time (Adjusted Wald F _2,3966_ = 168.99; *P* < .001). Survey-weighted estimated probabilities rose from 3.3% in 2019 to 6.9% in 2020 and peaked at 13.2% in 2021, before tapering to 9.7% in 2023. These results aligned with estimates from a model using quarter fixed effects (ie, indicator variables for each quarter), yielding nearly identical survey-weighted trajectories and supporting the spline specification as a parsimonious, interpretable representation of the underlying trend (eFigure and eTable in Supplement 1).

### Multivariable Factors Associated With CHS Diagnosis

Results from the multinomial logistic regression revealed several demographic and clinical factors associated with CHS diagnosis relative to other cannabis-related and vomiting-related presentations (Table 2). In adjusted models, males had higher odds of receiving a CHS diagnosis compared with females (RRR, 0.92; 95% CI, 0.88-0.95). Age was strongly associated with CHS, with patients aged 18 to 25 years over 3 times more likely to be diagnosed with CHS (RRR, 3.12; 95% CI, 2.90-3.35) compared with those aged 36 to 50 years, and those aged 26 to 35 years had similarly elevated odds (RRR, 2.84; 95% CI, 2.64-3.05).

**Table 2.** Multinomial Logistic Regression of Factors Associated With ED Visits^a^

	CUD only, RRR (95% CI)	*P* value	CVS only, RRR (95% CI)	*P* value	CHS, RRR (95% CI)	*P* value
Sex						
Male	1 [Reference]	NA	1 [Reference]	NA	1 [Reference]	NA
Female	0.55 (0.55-0.56)	<.001	1.23 (1.22-1.25)	<.001	0.92 (0.88-0.95)	<.001
Race and/or ethnicity						
White	1 [Reference]	NA	1 [Reference]	NA	1 [Reference]	NA
American Indian or Alaska Native	1.16 (1.02-1.31)	.02	1.00 (0.85-1.17)	.95	0.86 (0.66-1.12)	.26
Asian or Pacific Islander	0.45 (0.38-0.52)	<.001	0.85 (0.74-0.97)	.01	0.37 (0.31-0.44)	<.001
Black	1.52 (1.41-1.63)	<.001	0.96 (0.91-1.00)	.07	1.09 (1.02-1.17)	.008
Hispanic	0.74 (0.70-0.79)	<.001	0.87 (0.83-0.92)	<.001	0.66 (0.61-0.72)	<.001
Other^b^	0.88 (0.82-0.94)	<.001	0.87 (0.82-0.93)	<.001	0.70 (0.62-0.79)	<.001
Age category, y						
36-50	1 [Reference]	NA	1 [Reference]	NA	1 [Reference]	NA
12-17	1.01 (0.96-1.07)	.64	2.20 (2.00-2.42)	<.001	1.15 (1.04-1.28)	.008
18-25	2.09 (2.03-2.14)	<.001	2.17 (2.12-2.21)	<.001	3.59 (3.38-3.81)	<.001
26-35	1.61 (1.58-1.63)	<.001	1.51 (1.48-1.54)	<.001	2.26 (2.14-2.40)	<.001
51-64	0.50 (0.50-0.51)	<.001	0.74 (0.72-0.76)	<.001	0.30 (0.28-0.32)	<.001
≥65	0.10 (0.09-0.10)	<.001	0.63 (0.61-0.65)	<.001	0.05 (0.04-0.06)	<.001
Region						
Northeast	1 [Reference]	NA	1 [Reference]	NA	1 [Reference]	NA
Midwest	0.88 (0.76-1.01)	.07	0.83 (0.74-0.92)	<.001	0.69 (0.60-0.79)	<.001
South	0.72 (0.63-0.82)	<.001	0.85 (0.76-0.94)	.002	0.46 (0.40-0.53)	<.001
West	1.00 (0.86-1.15)	.97	1.04 (0.94-1.15)	.47	1.03 (0.89-1.19)	.73
ED event						
Treated and released	1 [Reference]	NA	1 [Reference	NA	1 [Reference]	NA
Admitted to same hospital	6.01 (5.6-6.42)	<.001	0.10 (0.09-0.10)	<.001	1.73 (1.59-1.89)	<.001
Transfer to short-term hospital	1.27 (1.19-1.35)	<.001	0.45 (0.42-0.48)	<.001	0.33 (0.26-0.41)	<.001
Died in ED	0.39 (0.33-0.46)	<.001	0.09 (0.06-0.14)	<.001	0.00 (0.00-0.00)	<.001
Not admitted, unknown destination	0.58 (0.38-0.88)	.01	2.77 (1.34-5.72)	.006	0.37 (0.18-0.75)	.006
Quarter (linear, centered at quarter 1 2019)	1.0 (1.01-1.1)	.02	1.02 (0.97-1.03)	.54	2.10 (1.94-2.27)	<.001
Quarter squared (quadratic)	1.00 (0.99-1.00)	.06	1.00 (0.99-1.00)	.52	0.98 (0.98-0.99)	<.001

Abbreviations: CHS, cannabinoid hyperemesis syndrome; CUD, cannabis use disorder; CVS, cyclic vomiting syndrome; ED, emergency department; NA, not applicable; RRR, relative risk ratio.

^a^ Estimates are from survey-weighted multinomial logistic regression using the Nationwide Emergency Department Sample, 2019 to 2022; sample restricted to age 12 years and older. Models compare CHS, CUD-only, and CVS-only with a reference group having neither diagnosis and account for the complex sampling design. Quarter represents a calendar quarter (linear term, centered at quarter 1 2019); quarter squared is a quadratic term capturing nonlinear trends.

^b^ The Healthcare Cost and Utilization Project breaks race and ethnicity into American Indian or Alaska Native, Asian or Pacific Islander, Black, Hispanic, Other, and White. Other includes additional or multiple races and includes no further information.

Racial and ethnic and regional disparities were also observed. Black patients had slightly higher odds of CHS diagnosis relative to White patients (RRR, 1.09; 95% CI, 1.02-1.17), whereas Hispanic (RRR, 0.66; 95% CI, 0.62-0.71) and Asian or Pacific Islander (RRR, 0.37; 95% CI, 0.31-0.45) patients had significantly lower odds. Regionally, CHS diagnoses were substantially less likely in the South (RRR, 0.46; 95% CI, 0.40-0.53) and Midwest (RRR, 0.69; 95% CI, 0.59-0.79) compared with the Northeast.

Hospital disposition was also associated with diagnostic classification. Patients admitted to the same hospital were significantly more likely to receive a CHS diagnosis than those treated and released (RRR, 1.73; 95% CI, 1.59-1.89), while those who were transferred or died in the ED had markedly lower odds. The probability of CHS diagnosis increased over time, as indicated by a significant positive linear trend (RRR for Q, 2.10; 95% CI, 1.94-2.27) and a quadratic term (: RRR for Q squared, 0.98; 95% CI, 0.98-0.99), suggesting a plateau in recent time periods.

## Discussion

This cross-sectional study documents a marked increase in ED visits consistent with CHS from 2016 to 2022, with the most pronounced rise occurring during the COVID-19 pandemic. Incidence was stable through 2019, rose sharply during 2020 to 2021, then declined modestly. Whereas cannabis-related diagnoses and CHS both exhibited sharp increases during 2020 and 2021, the rate of CVS diagnoses remained relatively stable. This divergence suggests that the increase in CHS may not reflect a general rise in emesis-related conditions, but rather a shift in the proportion of emesis cases attributable to cannabis use. Notably, the probability of receiving a CHS diagnosis among patients with a primary CVS diagnosis mirrored the broader rise in cannabis-related diagnoses, supporting the idea that increased cannabis exposure may be driving a growing subset of emesis cases. These results also align with evidence that legalization has facilitated access to high-potency cannabis, which is associated with acute harms, including CHS.^21,53^

The COVID-19 pandemic likely catalyzed the rise in CHS through stress, isolation, and increased cannabis use. After peaking in 2021, CHS incidence declined but plateaued above prepandemic levels, suggesting sustained structural or clinical drivers.^54,55,56^ The absence of a clear rise in CHS prior to 2020, despite expanding cannabis legalization and increasingly available high-potency products, presents a paradox. One possibility is underdiagnosis or misclassification before the syndrome gained broader clinical recognition. The post-2020 surge may thus reflect both increased exposure as well as increased diagnostic vigilance.

These findings underscore the importance of preparing ED clinicians and public health systems for the downstream consequences of increasing cannabis use, particularly with expanded access to high-potency consumables and in states where legalization is recent. CHS may be underrecognized in such environments, with failure to identify the condition leading to unnecessary testing and ineffective treatment as noted.^25^ Clinical guidelines and decision-support tools could aid clinicians in distinguishing CHS from other gastrointestinal conditions, especially in younger adults with chronic cannabis exposure.

### Limitations

This study had some limitations. Because CHS was not directly coded in *ICD-10* during the study period, we used proxy definitions, raising misclassification risk. Some cases may have been missed due to cannabis use underreporting, while others may have been misclassified due to overlapping symptoms. Requiring CVS as the primary diagnosis may also have underestimated case counts. To reduce diagnostic ambiguity, we excluded emesis visits potentially linked to pregnancy, migraines, or chemotherapy, possibly shifting CVS counts if coding of these alternative causes changed over time. NEDS captures visits, not patients, so repeat visits could inflate counts. Coding practices likely evolved with clinician familiarity and pandemic disruptions, affecting observed trends. Early 2020 coincided with widespread disruptions in ED care due to COVID-19, which likely reduced non–COVID-19 presentations and may have affected observed diagnostic patterns. Finally, race and ethnicity data were only available from 2019 to 2022, limiting trend analyses for this variable as well as the multinomial analyses to this truncated period.

## Conclusions

Despite these limitations, this cross-sectional study found that nationally representative ED data indicated a sustained rise in CHS-related visits from 2016 to 2022, especially among young adults. CHS now accounts for a growing share of emesis-related ED presentations. Given frequent misdiagnosis and costly, unnecessary testing, greater clinical awareness is needed. Targeted screening for cannabis use and recognition of symptom patterns could improve diagnostic accuracy. Although the new *ICD-10 *code might facilitate surveillance, its utility depends on clinician recognition. Validation studies to distinguish true incidence from coding shifts and misdiagnosis are also needed.

## Notes

Supplement 1. eMethods. Sensitivity Analysis Methods eTable. Information Criteria for Alternative Time Specifications (Non-Survey Weighted Models) eFigure. Temporal Pattern of CHS Under Alternative Time Specifications

Supplement 2. Data Sharing Statement
