# Supplementary material for: Cannabinoid Hyperemesis Syndrome, 2016 to 2022
Source: JAMA Netw Open. 2025 Nov 24;8(11):e2545310. doi: 10.1001/jamanetworkopen.2025.45310 (PMC12645340; doi:10.1001/jamanetworkopen.2025.45310)
Supplement: Supplement 1. — eMethods. Sensitivity Analysis Methods eTable. Information Criteria for Alternative Time Specifications (Non-Survey Weighted Models) eFigure. Temporal Pattern of CHS Under Alternative Time Specifications [file jamanetwopen-e2545310-s001.pdf]

Supplemental Online Content

Swartz JA, Franceschini D. Cannabinoid hyperemesis syndrome in the US, 2016 to 2022. *JAMA Netw Open.* 2025;8(11):e2545310.
doi:10.1001/jamanetworkopen.2025.45310

eMethods. Sensitivity Analysis Methods

eTable. Information Criteria for Alternative Time Specifications (Non-Survey Weighted Models)

eFigure. Temporal Pattern of CHS Under Alternative Time Specifications

This supplemental material has been provided by the authors to give readers additional information about their work.

© 2025 Swartz JA et al. *JAMA Network Open*.

This file contains additional analyses and supporting material referenced in the manuscript, *Cannabinoid Hyperemesis Syndrome in the US, 2016-2022*.

eMethods. Sensitivity Analysis Methods

We conducted sensitivity analyses to assess the robustness of temporal trend estimates for cannabinoid hyperemesis syndrome (CHS). In addition to the prespecified restricted cubic spline with knots at quarters 8, 15, 18, and 25 (corresponding to distinct phases of the COVID-19 pandemic), we estimated spline models with evenly spaced knots, percentile-based knots, and models with 3 and 5 knots. We also fit a quarter fixed-effects (FE) model, which estimates a separate parameter for each calendar quarter. As expected, the FE specification achieved lower information criteria due to its fully saturated nature but provided little interpretive parsimony. Across specifications, survey-weighted predicted probabilities of CHS were very similar, reinforcing that substantive conclusions were stable regardless of knot placement or model form.

Table e2 and **Figure e1** provide results of the sensitivity analyses of alternative time specifications for modeling CHS trends, including spline models with different knot placements and a quarter fixed-effects specification. These materials demonstrate robustness of the findings reported in the main manuscript.

eTable Information Criteria for Alternative Time Specifications (Non-Survey-Weighted Models)

Model	N	Log-likelihood	AIC	BIC
**COVID-knot spline**	412,991	-90369.589	180747.18	180790.9 |

© 2025 Swartz JA et al. *JAMA Network Open*.

**Evenly spaced spline**	412,991	-90456.829	180921.66	180965.38
**Percentile-knot spline**	412,991	-90557.909	181123.82	181167.54
**3-knot spline**	412,991	-91160.372	182326.74	182359.54
**5-knot spline**	412,991	-90532.055	181074.11	181128.77
**Quarter FE model**	412,991	-89959.885	179975.77	180281.84

Note: Displayed are N, log-likelihood, Akaike information criterion (AIC), and Bayesian information criterion (BIC) for the prespecified spline, alternative spline placements, and quarter fixed-effects specification.

eFigure Temporal Pattern of CHS Under Alternative Time Specifications

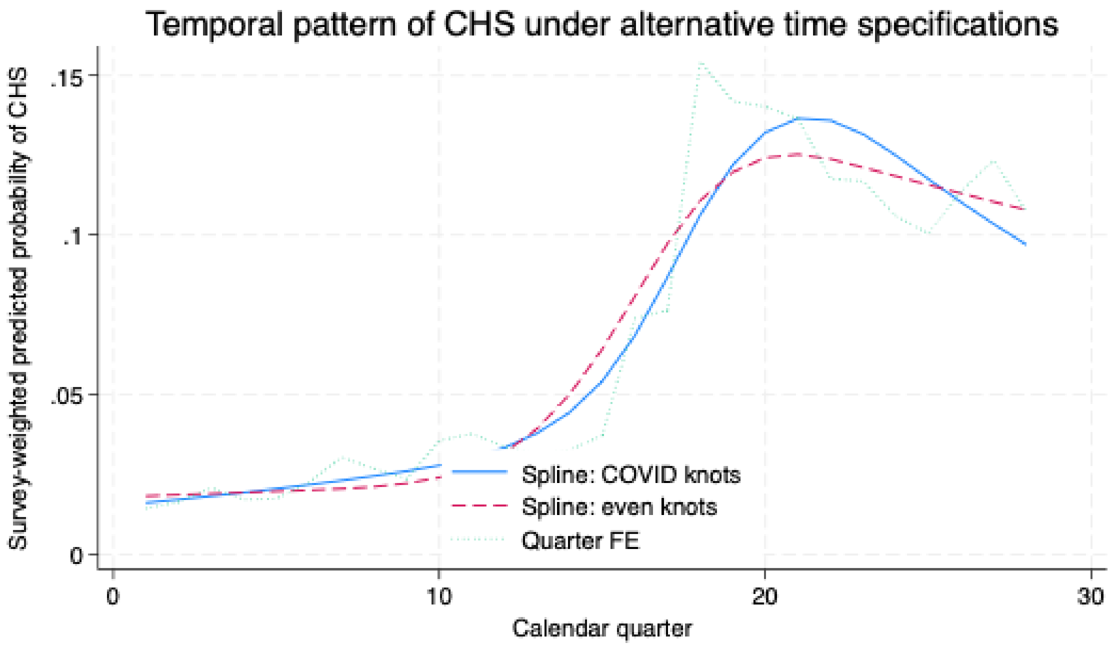

Note. Survey-weighted predicted probabilities of CHS from models using a restricted cubic spline with prespecified COVID-phase knots (quarters 8, 15, 18, 25), a spline with evenly spaced knots, and a quarter fixed-effects (FE) specification are shown. Predicted trajectories were nearly identical across specifications. The FE model, as expected, achieved the best fit but at the expense of parsimony, whereas the spline model provided a smoother, interpretable functional form.

© 2025 Swartz JA et al. *JAMA Network Open*.
