# Supplementary material for: Cannabinoid Hyperemesis Syndrome, 2016 to 2022
Source: JAMA Netw Open. 2025 Nov 24;8(11):e2545310. doi: 10.1001/jamanetworkopen.2025.45310 (PMC12645340; doi:10.1001/jamanetworkopen.2025.45310)
Supplement: Supplement 2. — Data Sharing Statement [file jamanetwopen-e2545310-s002.pdf]

# Data Sharing Statement

Swartz. Cannabinoid Hyperemesis Syndrome, 2016 to 2022. *JAMA Netw Open*. Published November 24, 2025. doi:10.1001/jamanetworkopen.2025.45310

**Data**
**Data available:** No

**Additional Information**
**Explanation for why data not available:** We used HCUP NEDS data. These data are maintained privately by HCUP but are available for purchase.
